# A survey of *Wolbachia* infection in brachyceran flies from Iran

**DOI:** 10.1371/journal.pone.0301274

**Published:** 2024-05-22

**Authors:** Ghazal Khosravi, Kamran Akbarzadeh, Fateh Karimian, Mona Koosha, Shahin Saeedi, Mohammad Ali Oshaghi

**Affiliations:** 1 Department of Vector Biology and Control of Diseases, School of Public Health, Tehran University of Medical Sciences, Tehran, Iran; 2 Department of Parasitology, Pasteur Institute of Iran, Tehran, Iran; Instituto Leonidas e Maria Deane Fiocruz Amazonia, BRAZIL

## Abstract

*Wolbachia* is a maternally inherited intracellular bacterium that is considered to be the most plentiful endosymbiont found in arthropods. It reproductively manipulates its host to increase the chances of being transmitted to the insect progeny; and it is currently used as a means of suppressing disease vector populations or controlling vector-borne diseases. Studies of the dissemination and prevalence of *Wolbachia* among its arthropod hosts are important for its possible use as a biological control agent. The molecular identification of *Wolbachia* relies on different primers sets due to *Wolbachia* strain variation. Here, we screened for the presence of *Wolbachia* in a broad range of Brachycera fly species (Diptera), collected from different regions of Iran, using nine genetic markers (*wsp*, *ftsZ*, *fbpA*, *gatB*, *CoxA*, *gltA*, *GroEL dnaA*, and *16s rRNA*), for detecting, assessing the sensitivity of primers for detection, and phylogeny of this bacterium. The overall incidence of *Wolbachia* among 22 species from six families was 27.3%. The most commonly positive fly species were *Pollenia* sp. and *Hydrotaea armipes*. However, the bacterium was not found in the most medically important flies or in potential human disease vectors, including *Musca domestica*, *Sarcophaga* spp., *Calliphora vicinia*, *Lucilia sericata*, and *Chrysomya albiceps*. The primer sets of *16s rRNA* with 53.0% and *gatB* with 52.0% were the most sensitive primers for detecting *Wolbachia*. Blast search, phylogenetic, and MLST analysis of the different locus sequences of *Wolbachia* show that all the six distantly related fly species likely belonging to supergroup A. Our study showed some primer sets generated false negatives in many of the samples, emphasizing the importance of using different loci in detecting *Wolbachia*. The study provides the groundwork for future studies of a *Wolbachia*-based program for control of flies.

## Introduction

Medically important flies belonging to the order Diptera, suborder Brachycera, and the families Sarcophagidae, Calliphoridae, Muscidae, and Fannidae are the vectors of many human and animal pathogens, causing significant nuisance and disease [1–5]. These insects have a worldwide spread, especially in the tropical and subtropical regions, and they cause many health problems and damage to livestock each year in those areas [1, 6]. In human societies, the fly families Muscidae, Calliphoridae, Sarcophagidae, and Fannidae live in close contact with humans [1–6], with adult flies and larvae feeding on human and pet feces, decomposing organic matter and spoiling meat [1, 7]. Moreover, several fly species cause myiasis, which is caused by the hatching and larval growth of the Cyclorrhapha fly in the human or animal body [8–10]. These flies have developed resistance to several different chemical insecticides, and the use of such insecticides can also contaminate the environment [2]. An alternative strategy for controlling the fly population is biological control. Recently, significant progress in our understanding of *Wolbachia* population ecology and genetics has reinforced the idea of using *Wolbachia* as a new method in the biological control of medically, veterinary, and agriculturally important arthropods [11–14]. Induction of cytoplasmic incompatibility (CI) by this endosymbiont is the most popular mode of reproductive alteration, in which females that are either uninfected or infected with a different strain of *Wolbachia* fail to develop viable eggs when cross with *Wolbachia*-infected males [15, 16]. Releasing male with *Wolbachia* suppresses vector population and hence vector-borne diseases [17]. Hence, it is essential to find new *Wolbachia* strains in flies that provide complete incompatibility.

*Wolbachia* was first observed and isolated by Marshall Hertig and S. Burt Wolbach in the reproductive tissue of *Culex pipiens* mosquitoes [18]; it is an obligate intracellular gram-negative bacterium and is classified in class Alphaproteobacteria, order Rickettsiales [18, 19]. This bacterium is transmitted vertically from the female insect to the next generation through the cytoplasm of the egg and can also be horizontally transmitted to other species [20, 21]. *Wolbachia* is a widespread intracellular bacterial symbiont that has been isolated from various insect species, ticks, and nematodes [20–22]. The *Wolbachia* genus has only one species, which has recently, based on gene sequence information, been classified into 21 major clades of *Wolbachia* known as ‘supergroups’ (A–F, H-Q, and S-W), with each group being classified into dozens of different subgroups, often based on analysis of the bacterial surface protein (*wsp*) [23–29]. Supergroups A and B are known to exclusively infect arthropods. The presence of this bacterium in the insect reproductive tissue is the result of changes in the host reproductive system that include cytoplasmic incompatibility (CI), thelytokous parthenogenesis, male feminization, and male killing [3, 30–33]. It is also reported that *Wolbachia* can play important roles in insect speciation and local adaptation [34].

It is known that different primer sets vary in their capability to detect *Wolbachia* in their hosts [35]. Different molecular markers such as *16s rRNA*, *ftsZ* and *wsp* have been widely used to detect and to study the phylogenetics of *Wolbachia* [28, 36], however, several investigations have shown the false negative for *ftsZ* [21, 37, 38] or false positive results for *16s rRNA* and *wsp* markers in discriminating between supergroups A and B which can lead to unreliable results in bacterial detection when these primer sets are used alone [39]. Besides, detection of recombination both between and within *Wolbachia* genes [40] suggests that a single-locus approach to strain characterization may be misleading [41].

*Wolbachia* has previously been detected in some Brachyceran flies, for example in *Hydrotea spinigena*, *Musca domestica*, and *Haematobia irritans irritans* of Muscidae and *Sarcophaga dux* of Sarcophagidae family [3, 38]. In Iran, there are some reports regarding the detection of *Wolbachia* in limited specific arthropod groups, but, so far, no general surveys of *Wolbachia* distribution among arthropods have been conducted [42]. For example, infection of *Wolbachia* has been detected in some species of *Phlebotomus* [22, 43–46], in cockroaches [47], and in some species of Diptera—in Culicidae [48, 49] and in Coleoptera (Staphilinidae) [50, 51]. However, the presence of *Wolbachia* in different fly species has not yet been studied. In the present study using nine different genetic markers, we show the incidence of *Wolbachia* infection in medically important flies of the suborder Brachycera (and one species of Nematocera), originating from a vast geographical area of Iran.

## Materials and methods

### Fly collection and identification

**Fig 1:**
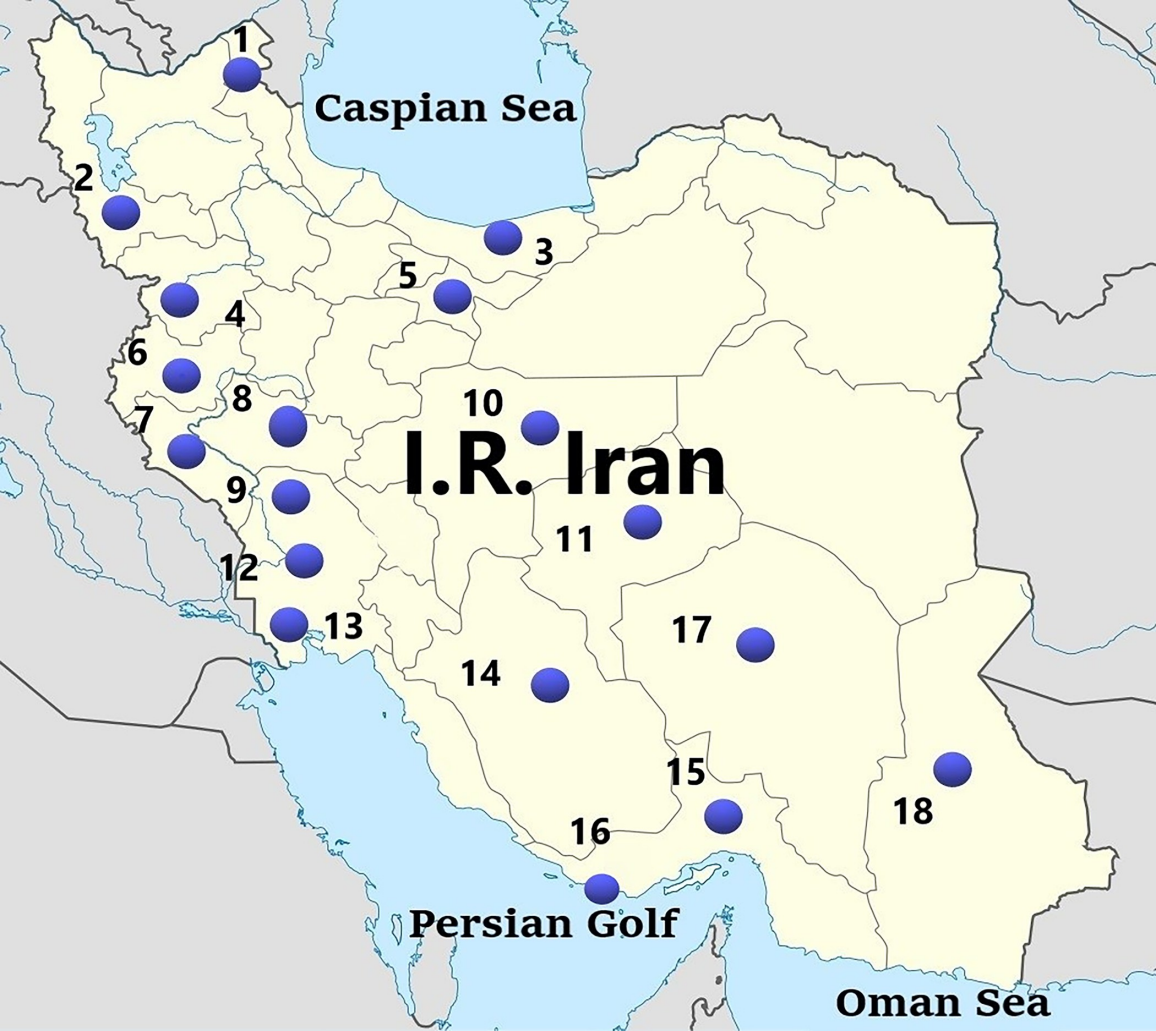
Map showing the locations (blue dots) where brachyceran flies were collected in this study. 1:Pars Abad, 2:Urmia, 3:Qaemshahr, 4:Sanandaj, 5: Tehran, 6: Kermanshah, 7: Ilam, 8: Alashtar, 9: Dezful, 10: Esfahan, 11: Yazd, 12:Hamidiyeh, 13: Abadan, 14: Kazeron, 15: Kish, 16:Bandar Abbas, 17:Kerman, 18:Zahedan. Reprinted from Choubdar et al 2021 [47] under a CC BY license, with permission from PLOS publisher, original copyright. (https://commons.wikimedia.org/wiki/File:Map_of_Iran.png).

Adult fly specimens were collected from various field sites covering the northern, western, southeastern, and central regions of Iran, from 2016 to 2021. Samples were obtained directly using insect netting over plants or garden beds, or traps already designed by Akbarzadeh et al [52] using liver baits including chicken (*Gallus gallus domesticus*), fish (*Oncorhynchus mykiss*), and beef (*Bos indicus*) based on their local availability, from urban, semi-urban, and rural settings in 18 different districts (Fig 1). Physically large fly (more than 0.5 cm in length) specimens were kept dry in silica (large samples) whereas small specimens were preserved in 70% ethanol and stored at –20°C until identification and DNA extraction. Specimens were identified according to the parameters of Crosskey and Lane [53], James [54], Zumpt [55] and Parchami-Araghi [56]. For sarcophagid flies, male specimens were morphologically identified to species level, based on the comparative morphology of male genitalia, whereas females were recorded as *Sarcophaga* sp. because it is not possible to identify them to species level morphologically.

#### DNA extraction and PCR

Whole bodies of small flies were homogenized individually in squash buffer [57], whereas larger flies were dissected in 1X PBS, and leg/s were used for homogenization and DNA extraction. Although reproductive tissues harbor more *Wolbachia*, it was more complex to retrieve DNA from the tiny reproductive tissues of dried specimens preserved for long time than legs. Total genomic DNA was extracted using the Collins method and kept at –20°C for further molecular investigations [57]. In case of a lack of proper DNA sample (poor DNA quality and quantity), the Qiagen DNA extraction kit was used, following the protocols recommended by the manufacturer (Qiagen, Hilden, Germany). Briefly, each specimen was homogenized in a special lysate buffering condition followed by QIAamp spin procedure which allows binding of the DNA to the QIAamp membrane upon centrifugation. Then the bound DNA was washed in two centrifugation steps and the purified DNA was eluted from the QIAamp spin column using a buffered solution. The DNA was eluted in 50–100μl buffer (based on the specimen size) and subsequently stored at 4°C. All samples were measured using a NanoDrop and quantified by spectrophotometer (Thermo Scientific™ NanoDrop™ One, MA, USA) at 260nm and diluted to a final concentration of 10 to 50 ng genomic DNA per μl. Barcode region of mitochondrial COI gene amplification served as an internal control to monitor the efficiency of both the DNA extraction and amplification [58].

To test the sensitivity of different primer sets, flies were screened for the presence of *Wolbachia* using standard PCR against nine different loci. Initial screening started with the primers *wsp*81F and *wsp*691R (Table 1) to amplify 632 bp of partial sequence of the *wsp* gene of *Wolbachia* [20, 59, 60]. Screening was followed by using other loci: *ftsZ*, *fbpA*, *gatB*, *CoxA*, *gltA*, *GroEL*, *16s rRNA*, and *dnaA*, using the primers and thermal program shown in Tables 1 and 2. Control DNA samples were prepared using double distilled water (ddH_2_O) as negative and previously confirmed adult females of either the *Culex pipens* mosquito [48] or the *Phlebotomus papatasi* sand fly [43] as positive controls, infected respectively with the *w*Pip or *w*Pap strains of *Wolbachia*. PCR amplifications were performed in an automatic thermocycler (Eppendorf, Germany) in a total volume of 25 μl containing 2–5 μl (~0.5 μg) of genomic DNA, 12.5 μL of Taq DNA Polymerase 2x Master Mix RED, Ampliqon (Denmark), 1 μL of each primer (10 mM), supplemented with ddH_2_O. PCR products were electrophoresed on 1% agarose gels with a voltage of 85 v and time less than 30 minutes, and the size of each PCR product was estimated using a 100-base pair (bp) molecular marker (SinaClon, Iran), visualized under a UV transilluminator. Samples with expected PCR product sizes as indicated in Table 1 and lack of PCR products were considered as positive and negative to *Wolbachia* respectively. For samples that did not amplify, conventional PCRs were repeated.

**Table 1:** List of the primers and loci used for detection, identification, and phylogeny of *Wolbachia* in this study.

No	Gene/locus	Primer 5’-3’	PCR product size (bp)	References
1	***wsp***	*wsp*81F: TGGTCCAATAAGTGATGAAGAAAC *wsp*691R: AAAAATTAAACGCTACTCCA	632	[20]
2	***FtsZ***	*ftsZ*qPCRF: CATTGCAGAGCTTGGACTT *ftsZ*qPCR R: TCTTCTCCTTCTGCCTCTCC	586–775	[67–71]
3	***GroEL***	WgroF1d: GGT GAG CAG TTR CAR SAA GC WgroRev1d: AGRTCTTCCATYTTRATTCC	450–500	[68, 69]
4	***CoxA***	*CoxA*_F1: TTGGRGCRATYAACTTTATAG *CoxA*_R1: CTAAAGACTTTKACRCCAGT	450–500	[68, 69]
5	***fbpA***	*fbpA*_F1: GCTGCTCCRCTTGGYWTGAT *fbpA*_R1: CCRCCAGARAAAAYYACTATTC	450–500	[68, 69]
6	***dnaA***	*dnaA*2F: ACAATTGGTTATATCAGCTG *dnaA*2R: TACATAGCTATTTGYCTTGG	535	[69]
7	***gatB***	*gatB*_F1: AKTTAAAYCGYGCAGGBGTT *gatB*_R1: GGYAAYTCRGGYAAAGATGA	693	[67, 68]
8	***gltA***	W*gltA*F1: TACGATCCAGGGTTTGTTTCTAC W*gltA*Rev2: CATTTCATACCACTGGGCAA	450–500	[68, 69]
9	***16s rRNA***	15F: GTGCCAGCMGCCGCGGTAA 806: GGACTACHVGGGTWTCTAA	450–500	[21]

**Table 2:** The PCR thermal programs used for amplification of different *Wolbachia* loci.

Gene/locus program	*wsp*	*FtsZ*	*GroEL*		*CoxA*	*fbpA*	*dnaA*	*gatB*	*gltA*	*16S rRNA*
**Preheating(°C)**	95	95	94		94	94	94	94	94	94
**Time (min)**	4	5	2		5	5	5	2	2	3
**No. of Cycles**	30	36	5*	34**	36	36	30	36	40	35
**Denaturation(°C)**	94	95	94	94	94	94	94	94	94	94
**Time (sec)**	45	30	45	45	15	30	60	30	45	45
**Annealing (°C)**	58	51	60	55	55	59	50	56	53	50
**Time (sec)**	45	30	45	45	15	45	60	45	45	60
**Extension (°C)**	72	72	72	72	72	72	72	72	72	72
**Time (sec)**	40	60	80	80	30	90	120	90	90	80
**Final extension(°C)**	72	72		72	72	72	72	72	72	72
**Time (min)**	4	5		10	10	10	10	10	10	10

*: first cycle

**: second cycle for *GroEL*

To perform phylogenetic analyses and to confirm the PCR results and, thereby, the *Wolbachia* infection status, a subset of 13 PCR products were sequenced. The PCR products with a clean, sharp, and single band with no smears and the expected sizes (Table 1) were selected for sequencing. The positive PCR products of *16s rRNA* (n = 4), *gatB* (n = 3), GroEL (n = 2), *wsp* (n = 1), *gltA* (n = 1), *dnaA* (n = 1), and *CoxA* (n = 1) genes were purified and sequenced bidirectionally by the Sanger analysis (Sinuhebiotech, Iran) using the same PCR primers used for PCR amplification. The sequences acquired in this study were edited and assembled using Chromas [61] software to construct consensus sequences by matching the forward and reverse sequences of the same PCR products to obtain an actual sequence which approximately occurs in the specimen genome. The sequence data were then analyzed using the NCBI Blast database (Nucleotide collection) [62]. *Wolbachia* classification was performed based on the identity score of the obtained sequences with the available GenBank sequences through the BLAST algorithm and phylogenetic analysis. Moreover, the sequences were compared with the data from the *Wolbachia* MLST database (https://pubmlst.org/bigsdb?db=pubmlst_*Wolbachia*_seqdef). All the sequences obtained were submitted to GenBank database [63]. The consensus high-confidence sequences were aligned with a subset of other corresponding *Wolbachia* sequences that were available in GenBank, using multiple-sequence alignments available in CLUSTAL Omega [64].

### Phylogenetic analysis

For phylogenetic analysis, we combined the *Wolbachia* sequences obtained in this study with a subset of the representative sequences of *Wolbachia* from different supergroups, available in GenBank (Table 3). All the DNA sequences (S1 Fig) used for alignment were cut to obtain a consistent region. The data were aligned (S2 Fig), and the Neighbor-Joining (NJ) algorithm distance analysis using the Kimura 2- parameter model [65] was employed to construct a phylogenetic tree with bootstrap analysis of 1000 replicates in MEGA 7 software [66].

**Table 3:** Details (host classification, supergroups, and GenBank ID) of *16s rRNA* gene sequences of *Wolbachia* strains used for phylogenetic analysis in this study.

Host	Family (Order)	Supergroup	GenBank ID	Reference
***Delia platura***	Anthomyiidae	A	OR793855	This study
***Hydrotaea armipes***	Muscidae	A	OR793856	This study
***Fannia canicularis***	Fannidae	A	OR793857	This study
***Pollenia*** sp.	Calliphoridae (Dip.)	A	OR793858	This study
***Anoplius nigerrimus***	Pompilidae (Hym.)	A	OX366385	DS
***Calamotropha paludella***	Crambidae (Lep.)	A	OX366397	DS
***Matsuccocus pini***	Matsucoccidae (Hem.)	A	MK462264	DS
***Ixodes ricinus***	Ixodidae (Ixo.)	A	MW727243	DS
***Drosophila santomea***	Drosophilidae (Dip.)	A	CP069060	DS
***Pheosia gnoma***	Notodontidae (Lep.)	A	OX366379	DS
***Cinara cedri***	Aphididae (Hem.)	B	JN384059	[72]
***Nilaparvata lugens***	Delphacidae (Hem.)	B	GU124506	[73]
***Philaenus maghresignus***	Aphrophoridae (Hem.)	B	AB772263	[74]
***Onchocerca gibsoni***	Onchocercidae (Rha.)	C	AJ276499	DS
***Brugia malayi***	Onchocercidae (Rha.)	D	AJ010275	[75]
***Folsomia candida***	Isotomidae (Coll.)	E	AF179630	[76]
***Kalotermes flavicollis***	Kalotermitidae (Bla.)	F	Y11377	[70]
***Mansonella ozzardi***	Onchocercidae (Rha.)	F	AJ279034	DS
***Folsomides parvulus***	Isotomidae (Coll.)	H	KT799586	[77]
***Orchopeas leocopus***	Ceratophyllidae (Sip.)	I	AY335924	[78]
***Myodopsylla gentilis***	Ischnopsyllidae (Sip.)	I	AY335918	[78]
***Dipetalonema gracile***	Onchocercidae (Fil.)	J	AJ548802	[79]
***Bryobia*** sp.	Tetranychidae (Acari)	K	EU499316	[80]
***Radopholus similis***	Pratylenchidae (Tyl.)	L	KF059257	DS
***Maculolachnus submacula***	Aphididae (Hem.)	M	JN384077	[72]
***Aulacorthum solani***	Aphididae (Hem.)	M	JN384087	[72]
***Porcellio dilatatus***	Porcellionidae (Iso.)	N	KJ814212	[78]
***Porcellio laevis***	Porcellionidae (Iso.)	N	KJ814213	[78]
***Bemisia tabaci***	Aleyrodidae (Hem.)	O	LN829671	DS
***Torotrogla merulae***	Syringophilidae (Acari)	P	KP114103	[81]
***Torotrogla cardueli***	Syringophilidae (Acari)	Q	KP114101	[82]
***Spinturnix*** sp.	Spinturnicidae (Acari)	U	KP165041	DS
***Spinturnix*** sp.	*Spinturnicidae* (Acari)	U	KP165042	DS
***Rickettsia*** sp.	Rickettsiaceae (Alphaproteobacteria)	Outgroup	AB795333	[83]

DS: Direct submission to GenBank database, Dip.: Diptera, Hym.: Hymenoptera, Rha.: Rhabditida (nematodes), Iso.: Isopoda, Hem.: Hemiptera, Lep.: Lepidoptera, Bla: Blattodea, Sip: Siphoneptra, Fil: Filarioidea (nematodes), Tyl: Tylenchida (nematodes), Ixo: Ixodida, Coll: Collembola

### Statistical analysis

The data obtained for sensitivity of different primer sets were analyzed with the aid of the one-way ANOVA test. All analyses were performed using the Graph Pad Prism 5.0 program, with a significance level of 5%.

### Ethic statement

This study followed the guidelines of the institutional ethical committee (Tehran University of Medical Sciences, TUMS). The protocols were approved by TUMS ethical committee under registry IR.TUMS.SPH.REC.1398.052.

## Results

In total, 1202 brachyceran fly specimens were collected, including 525 (43.7%) males and 677 (56.3%) females, from 22 species of six families: Muscidae, Calliphoridae, Sarcophagidae, Fannidae, Milichiidae, and Anthomyiidae (Table 4). The largest group were Muscidae (n = 519; 43.18%), followed by Calliphoridae (n = 489; 40.68%), Sarcophagidae (n = 108; 8.98%), Fannidae (n = 78; 6.49%), Milichiidae (n = 6, 0.5%), and Anthomyiidae (n = 1; 0.0008%). In addition to brachyceran flies, six moth fly specimens (*Psychoda* sp.) of Psychodidae belonged to Nematocera suborder were captured among the collected specimens.

**Table 4:** Location, geographical coordinate, classification, and gender of fly specimens collected in this study. *M*: *Musca*, *Mu*: *Muscina*, *F*: *Fannia*, *S*: *Sarcophaga*, *L*: *Lucilia*, *Ch*: *Chrysomya*, *H*: *Hydrotaea*, *C*: *Calliphora*, *D*: *Delia*, *St*: *Stomoxys*.

Province (District)	Date	Coordinate	Family	Species	Male	Female	Total
**Ardabil (Pars Abad)**	2019–04	39°38′36″N, 47°54′ 46″ E	Muscidae	*M*. *domestica*	31	26	57
		39°38′36″N, 47°54′46″ E		*Mu*. *stabulans*	0	12	12
		39°38′36″N, 47°54′ 46″ E	Calliphoridae	*Pollenia* sp.	35	25	60
		39°38′36″N, 47°54′ 46″ E	Fannidae	*F*. *canicularis*	23	2	25
		39°38′36″N, 47°54′ 46″ E	Sarcophagidae	*Sarcophaga* sp.	0	17	17
		39°38′36″N, 47°54′ 46″ E		*S*. *variegata*	11	0	11
**Mazandaran (Qaemshahr)**	2016–06	36°27′49″N, 52°51′ 29″ E	Muscidae	*M*. *domestica*	5	9	14
		36°27′49″N, 52°51′ 29″ E		*Muscina* sp.	0	2	2
		36°27′49″N, 52°51′ 29″ E		*Hydrotaea* sp.	1	0	1
		36°27′49″N, 52°51′ 29″ E	Calliphoridae	*L*. *sericata*	5	7	12
		36°27′49″N, 52°51′ 29″ E		*Ch*. *albiceps*	4	8	12
**Tehran (Tehran)**	2017–04	35°44′11″N, 51°16′ 23″ E	Muscidae	*M*. *domestica*	32	96	128
		35°44′11″N, 51°16′ 23″ E		*Mu*. *stabulans*	5	0	5
		35°44′11″N, 51°16′ 23″ E		*H*. *armipes*	2	0	2
		35°44′11″N, 51°16′ 23″ E	Calliphoridae	*L*. *sericata*	8	16	24
		35°44′11″N, 51°16′ 23″ E		*Ch*. *albiceps*	25	78	103
		35°44′11″N, 51°16′ 23″ E		*C*. *vicina*	63	47	110
		35°44′11″N, 51°16′ 23″ E		*Pollenia* sp.	0	3	3
		35°44′11″N, 51°16′ 23″ E	Fannidae	*Fannia* sp.	10	36	46
		35°44′11″N, 51°16′ 23″ E	Sarcophagidae	*S*. *variegata*	5	0	5
		35°44′11″N, 51°16′ 23″ E		*S*. *africa*	5	0	5
		35°44′11″N, 51°16′ 23″ E		*S*. *aegyptica*	4	0	4
		35°44′11″N, 51°16′ 23″ E		*S*. *dux*	1	0	1
		35°44′11″N, 51°16′ 23″ E		*Sarcophaga* sp.	0	8	8
		35°44′11″N, 51°16′ 23″ E	Psychodidae*	*Psychoda* sp.	3	3	6
		35°44′11″N, 51°16′ 23″ E	Milichiidae	*Desmometopa varipalpis*	3	3	6
		35°44′11″N, 51°16′ 23″ E	Anthomyiidae	*D*. *platura*	1	0	1
**Esfahan (Esfahan)**	2018–04	32°39’41’’N 51°40’49’’E	Muscidae	*M*. *domestica*	10	14	24
		32°39’41’’N 51°40’49’’E	Calliphoridae	*C*. *vicina*	3	11	14
**Yazd (Yazd)**	2019–06	31°53’51’’N 54°21’ 25’’ E	Muscidae	*M*. *domestica*	6	6	12
		31°53’51’’N 54°21’ 25’’ E		*St*. *calcitrans*	0	6	6
		31°53’51’’N 54°21’ 25’’ E	Calliphoridae	*Pollenia* sp.	0	6	6
**Fars (Kazeron)**	2017–4	29°36′56″N, 51°39′24″E	Muscidae	*M*. *domestica*	5	5	10
		29°36′56″N, 51°39′24″E	Calliphoridae	*L*. *sericata*	0	10	10
		29°36′56″N, 51°39′24″E		*Ch*. *albiceps*	0	5	5
		29°36′56″N ,51°39′24″E		*C*. *vicina*	10	5	15
		29°36′56″N ,51°39′24″E	Sarcophagidae	*Sarcophaga* sp.	0	10	10
**Hormozgan** **(Bandar Abbas)** **(Kish)**	2019–03	27°11′46″N, 56°17′16″E	Muscidae	*M*. *domestica*	6	8	14
		27°11′46″N, 56°17′16″E	Calliphoridae	*L*. *sericata*	7	0	7
		27°11′46″N, 56°17′16″E		*Ch*. *albiceps*	7	19	26
		27°11′46″N, 56°17′16″E		*Ch*. *marginalis*	0	14	14
		27°11′46″N ,56°17′16″E		*Ch*. *megacephala*	7	6	13
		27°11′46″N, 56°17′16″E	Fannidae	*Fannia* sp.	0	7	7
		27°11′46″N, 56°17′16″E	Sarcophagidae	*S*. *dux*	12	0	12
	2020–03	26°32′0″N, 53°58′0″E	Muscidae	*M*. *domestica*	11	13	24
**Urmia (Urmia)**	2019–05	37°33’9.9"N, 45°4’33.8"E	Muscidae	*M*. *domestica*	43	15	58
**Kurdistan (Sanandaj)**	2019–05	35°18′41″N, 46°59′46″E	Muscidae	*M*. *domestica*	7	6	13
		35°18′41″N, 46°59′46″E		*St*. *calcitrans*	5	12	17
		35°18′41″N, 46°59′46″E	Calliphoridae	*C*. *vicina*	0	13	13
**Kermanshah (Kermanshah)**	2019–04	34°19′57″N 47°05′36″E	Sarcophagidae	*S*. *variegata*	7	0	7
		34°19′57″N 47°05′36″E		*S*. *bellae*	7	0	7
		34°19′57″N 47°05′36″E		*S*. *aegyptica*	7	0	7
**Lorestan (Alashtar)**	2019–03	33°51′55″N, 48°15′44″E	Calliphoridae	*C*. *vicina*	0	14	14
**Sistan & Baluchistan (Zahedan)**	2020–03	29°30′9″N, 60°51′21″E	Muscidae	*M*. *domestica*	7	6	13
**Kerman (Kerman)**	2021–02	30°17′28″N 57°04′04″E	Muscidae	*M*. *domestica*	14	7	21
		30°17′28″N 57°04′04″E	Calliphoridae	*C*. *vicina*	11	3	14
		30°17′28″N 57°04′04″E		*L*. *sericata*	5	9	14
		30°17′28″N 57°04′04″E	Sarcophagidae	*Sarcophaga* sp.	0	14	14
**Khuzestan (Hamidiyeh) (Dezful)** **(Abadan)**	2020–03	31°28′52″N 48°26′06″E 32°22′43″N 48°24′52″E 30°20’21"N,48°18’15"E	Muscidae	*M*. *domestica*	13 11 11	9 7 10	22 18 21
**Ilam (Ilam)**	2020–04	33°38′06″N 46°24′26″E	Muscidae	*M*. *domestica*	14	12	26
**Total**			**7**	**23**	**528**	**680**	**1208**

*: Moth fly or sink fly from Nematocera suborder.

The highest number of specimens at species level were *M*. *domestica* (n = 475; 39.52%), followed by *C*. *vicina* (n = 180; 14.97%), *Pollenia* sp. (n = 69; 5.74%), *L*. *sericata* (n = 67, 5.57%), *Fannia* sp. (n = 53, 4.40%), *Sarcophaga* sp. (n = 49, 4.07%), and *Ch*. *albiceps* (n = 43, 3.58%) (Table 1). The remaining species had less than a three percent frequency rate.

Out of 1202 brachyceran flies, a total of 838 fly specimens (70%), including 363 males (69.1% of total males) and 475 females (70.2% of total females), were screened for *Wolbachia* in the 22 species. The results showed that out of the 838 specimens screened, only 66 specimens (7.88%), belonging to six species: *Fannia canicularis*, *Fannia* sp., *Hydrotaea armipes*, *Pollenia* sp., *Desmometopa varipalpis*, and *Delia platura*, were positive for *Wolbachia* (Table 5). The other 772 (92.12%) screened specimens, belonging to the remaining 16 species, were negative for the *Wolbachia* genome using the nine different primer sets. Also, the six moth fly (sink fly) specimens were free of *Wolbachia*.

**Table 5:** The sensitivity rate of different primer sets used for detection of *Wolbachia* in six brachyceran fly species, as determined by PCR assay against nine loci. The rate of positivity for each primer set is shown in the relevant cell.

Species	No of tested	No of Infected (%)	*Wsp* %	*ftsZ* %	*fbpA* %	*gatB* %	*CoxA* %	*gltA* %	*GroEL* %	*16sr* *RNA* %	*dnaA* %
***H*. *armipes***	2	2(100)	50	0	0	100	0	0	0	100	0
***Fannia*** sp.	15	10(66.7)	37.5	70	0	50	0	0	0	25	0
***F*. *canicularis***	25	17(68)	75	72	0	85	0	0	0	33	0
***Pollenia*** sp.	69	30(43.5)	48	65	70	77	93	75	63	60	80
***Delia platura***	1	1(100)	0	0	0	0	0	0	0	100	0
***Desmometopa varipalpis***	6	6(100)	0	0	0	0	0	0	0	0	100
**Total** **Mean %**	118	66 (55.93)	35.1	34.5	11.7	52.0	15.5	12.5	10.5	53.0	30.0

Overall, *Wolbachia* was found in 6 (27.3%) of the 22 brachyceran fly species surveyed, in which 37 (50.7%) of male specimens (n = 73) and 29 (64.4%) of female specimens (n = 45) of the six infected species were positive (Table 6). *Wolbachia* was found in six species: *Fannia canicularis* (n = 25, 68%), *Fannia* sp. (n = 15, 66.7%), *Hydrotaea armipes* (n = 2, 100%), *Pollenia* sp. (n = 69, 43.5%), and *Delia platura* (n = 1, 100%). The infection rate in females (64.4%) was found to be higher than that in males (50.7%) (Table 6).

**Table 6:** *Wolbachia*-infected brachyceran fly species from Iran based on sex that were screened using 9 sets of primers.

Species	Family	N.	Male (M)	Female (F)	*Wolbachia* positive		Infection Rate (%)	
					M	F	M	F
***Hydrotaea armipes***	Muscidae	2	2	0	2	0	100	NA
***Fannia*** sp.	Fannidae	15	9	6	7	3	77.7	50.0
***Fannia canicularis***	Fannidae	25	23	2	16	1	69.5	50.0
***Pollenia*** sp.	Calliphoridae	69	35	34	8	22	22.9	64.7
***Delia platura***	Anthomyiidae	1	1	0	1	0	100	NA
***Desmometopa varipalpis***	Milichiidae	6	3	3	3	3	100	100
***Total*** (mean %)	5	118	73 61.9	45 38.1	37 50.7	29 64.4		

*Wolbachia* DNA was not detected in 16 fly species: *Musca domestica*, *Muscina stabulans*, *Muscina* sp., *Sarcophaga* sp., *S*. *variegata*, *S*. *africa*, *S*. *aegyptica*, *S*. *dux*, *S*. *bellae*, *Hydrotaea* sp., *Luculia sericata*, *Chrysomya albiceps*, *Ch*. *marginalis*, *Ch*. *megacephala*, and *Calliphora vicina*.

**Fig 2:**
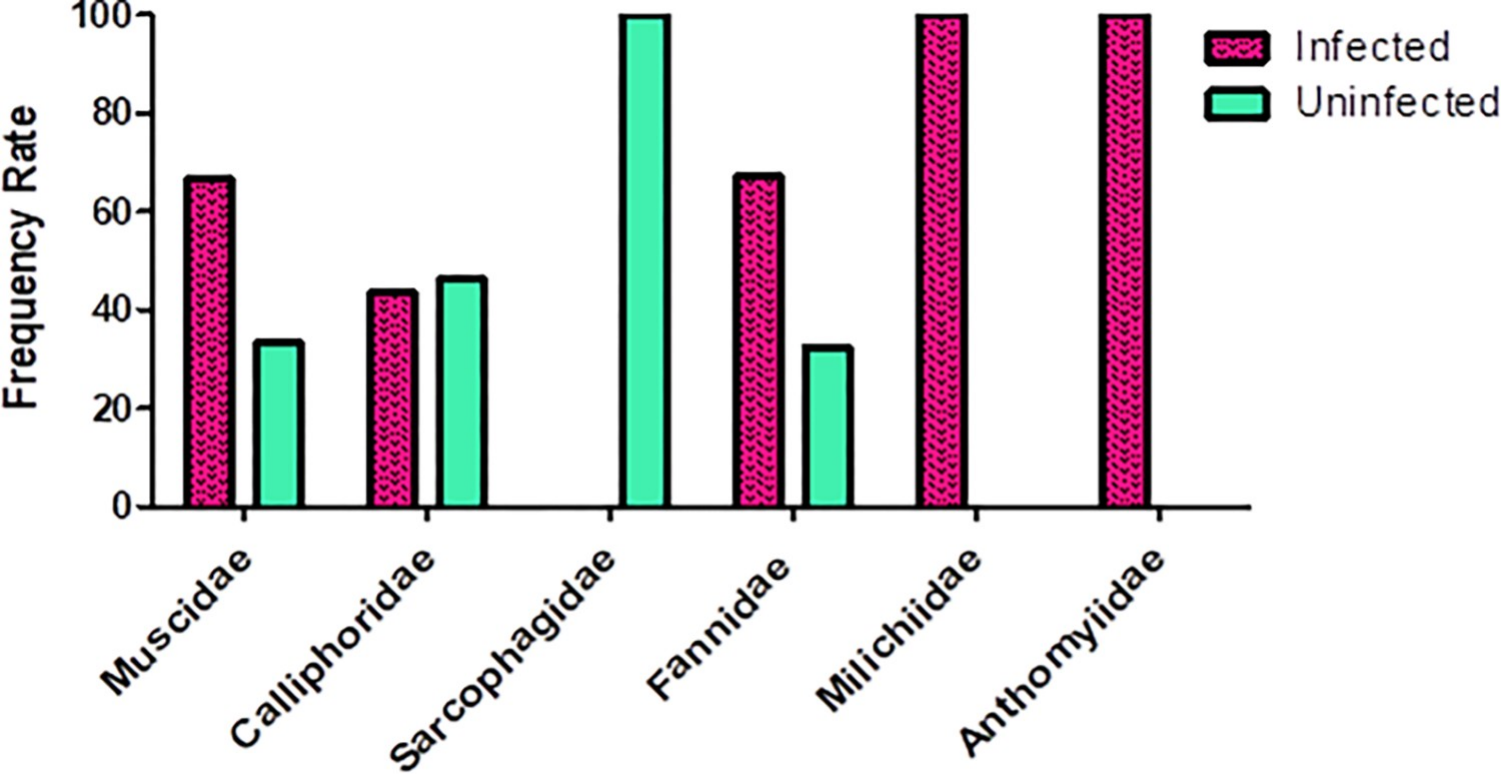
The proportion of species infected with *Wolbachia* in six brachycera families in Iran as determined by PCR assays against *16s rRNA*, *gatB*, *wsp*, *FtsZ*, *fbpA*, *CoxA*, *gltA*, *GroEL*, and *dnaA* loci.

Within the six families screened, no *Wolbachia* was detected in Sarcophagidae (six species tested). The families with the largest number of *Wolbachia*-infected species were Fannidae (n = 2 infected species; 67.5% infection), Muscidae (2; 66.7%), Calliphoridae (1; 43.5%), Milichiidae (1; 100%), and Anthomyiidae (1; 100%) (Fig 2).

**Fig 3:**
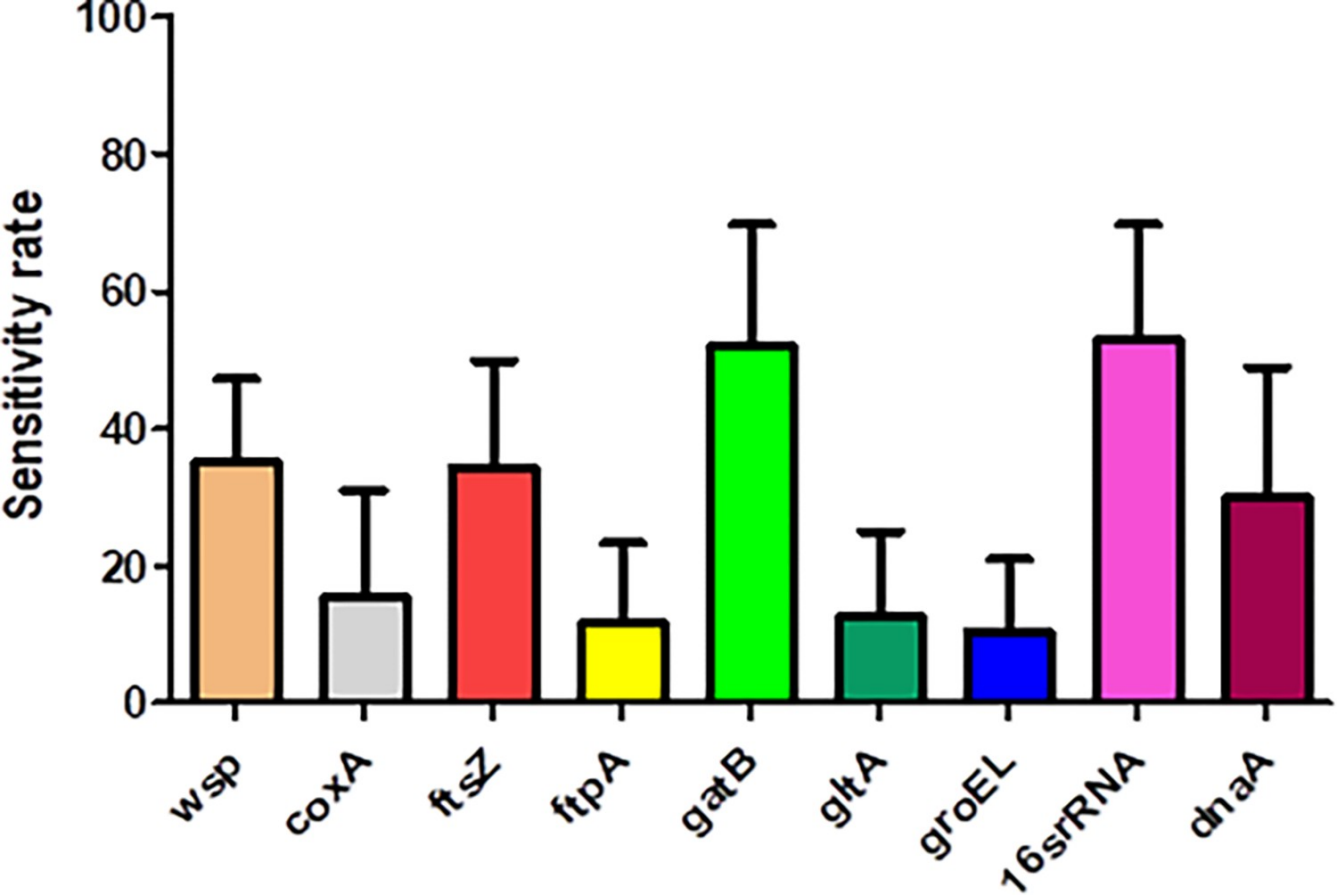
Sensitivity rate of the nine pairs of primers in detecting *Wolbachia* infection in six Iranian brachyceran fly species: *Fannia canicularis* (n = 25 with 68% infection rate), *Fannia* sp. (n = 15, 66.7%), *Hydrotaea armipes* (n = 2, 100%), *Pollenia* sp. (n = 69, 43.5%), *Desmometopa varipalpis* (n = 6, 100%), and *Delia platura* (n = 1, 100%). Bars show mean ± SEM, p>0.05, one-way ANOVA test).

In this study nine pairs of primers were used, to test the sensitivity of different primer sets as well as to increase the chance of detecting different *Wolbachia* strains in the fly specimens: *16s rRNA*, *gatB*, *wsp*, *ftsZ*, *fbpA*, *CoxA*, *gltA*, *GroEL*, and *dnaA*. PCR-amplified fragments were identical in length to the expected sizes (632 bp for *wsp*, 650–700 bp for *ftsZ*, 450–500 bp for *GroEL*, 450–500 bp for *CoxA*, 450–500 bp for *fbpA*, 535 bp for *dnaA*, 693 bp for *gatB*, 450–500 bp for *gltA*, and 450–500 bp for *16s rRNA)* (Table 1). Of the different primer sets, the *16s rRNA*, *gatB*, *wsp*, *ftsZ*, and *dnaA* primers in order were more sensitive in detecting *Wolbachia* infections than the other four primer sets, which were used for standard PCR. We found that, within the infected species, a mean of 53.0% specimens were positive for the *16s rRNA*, 52.0% for *gatB*, 35.1% for *wsp*, 34.5% for the *ftsZ*, and 30% for the *dnaA* primer set (Fig 3, Table 5). However, the differences between these five primer sets were not statistically significant (p>0.05, one-way ANOVA test).

### Sequence and phylogenetic analysis

The *Wolbachia* infection was confirmed by sequencing a subset of PCR-positive samples, as the Blasted sequences had several hits to *Wolbachia*. The Blast search showed the sequences obtained in this study had 98.36–100% similarities to the closest GenBank sequences of *16s rRNA*, *gltA*, *wsp*, *dnaA*, *GroEL*, *gatB*, and *CoxA* genes of *Wolbachia* strains (Table 7).

**Table 7:** Details of the *Wolbachia* strains detected in the Iranian brachyceran flies for different genes compared with GenBank entries.

Gene	Host fly	GenBank ID	Closest relatives according to BLASTn		
			Identity %	GenBank ID	Host (Order)
***16s rRNA***	*Delia platura*,	OR793855	100	CP037426	*Haematobia irritans* (Dip.) and >100 entries
	*Hydrotaea armipes*	OR793856	100	CP042904	*Drosophila ananassae* (Dip.) and >100 entries
	*Fannia canicularis*	OR793857	99.73	KJ728755	*Anopheles gambiae* (Dip.)
	*Pollenia* sp.	OR793858	100*	OQ102145 OX366388 OX366378 OX366395	*Hyalesthes luteipes* (Hem.) *Philonthus cognatus* (Col.) *Ectemnius continuus* (Hym.) *Bombylius major* (Dip.)
***wsp***	*Hydrotaea armipes*	OR865323	99.83	CP041215 OX366397 OX366353 OX366352 OX366332 CP054598 AB024571	*Carposina sasakii* (Lep.) *Calamotropha paludella* (Lep.) *Yponomeuta plumbellus* (Lep.) *Endotricha flammealis* (Lep.) *Cheilosia soror* (Dip.) *Ceratosolen solmsi* (Hym.) *Ephestia cautella* (Lep.)
***gatB***	*Hydrotaea armipes*	OR865320	99.10 99.10	KJ174696 EU127667	*Lutzomyia stewartia* (Dip.) *Pheidole obtusospinosa* (Hym.)
	*Pollenia* sp.	OR865321	99.10 99.10	KJ174696 EU127667	*Lutzomyia stewartia* (Dip.) *Pheidole obtusospinosa* (Hym.)
	*Fannia canicularis*	OR865322	99.10 99.10	KJ174696 EU127667	*Lutzomyia stewartia* (Dip.) *Pheidole obtusospinosa* (Hym.)
***coxA***	*Pollenia sp*.	OR865324	98.36 98.36	OX366380 OU909470	*Tiphia femorata* (Hym.) *Psylliodes chrysocephala* (Col.)
***gltA***	*Pollenia sp*.	OR865325	99.89	OZ014601	*Nomada hirtipes* (Hym.) and >12 entries
***groEL***	*Pollenia sp*.	OR865326	99.38	OX366395	*Bombylius major* (Hym.)
	*Pollenia sp*.	OR865327	99.38	OX366395	*Bombylius major* (Hym.)
***dnaA***	*Pollenia sp*.	OR865328	99.76	CP037426	*Haematobia irritans* (Dip.) and > 76 entries

*: There were 22 sequence entries in GenBank with 100% identity. Here, only one entry from each insect order is shown. Dip.: Diptera, Hem.: Hemiptera, Col.: Coleoptera, Hym.: Hymenoptera, Lep.: Lepidoptera.

The sequences obtained in this study have been submitted to GenBank with the Accession Numbers (GenBank IDs: OR793855-OR793858 correspond to *16s rRNA* gene of *Delia platura*, *Hydrotaea armipes*, *Fannia canicularis*, and *Pollenia sp*. respectively, and OR865320-OR865322 relate to *gatB* gene of *Hydrotaea armipes*, *Pollenia sp*., and *Fannia canicularis* respectively, OR865323 relates to *wsp* gene of *Hydrotaea armipes*, and OR865324-OR865328 correspond respectively to *coxA*, *gltA*, *groEL* (n = 2), and *dnaA* genes of *Pollenia sp*..

**Fig 4:**
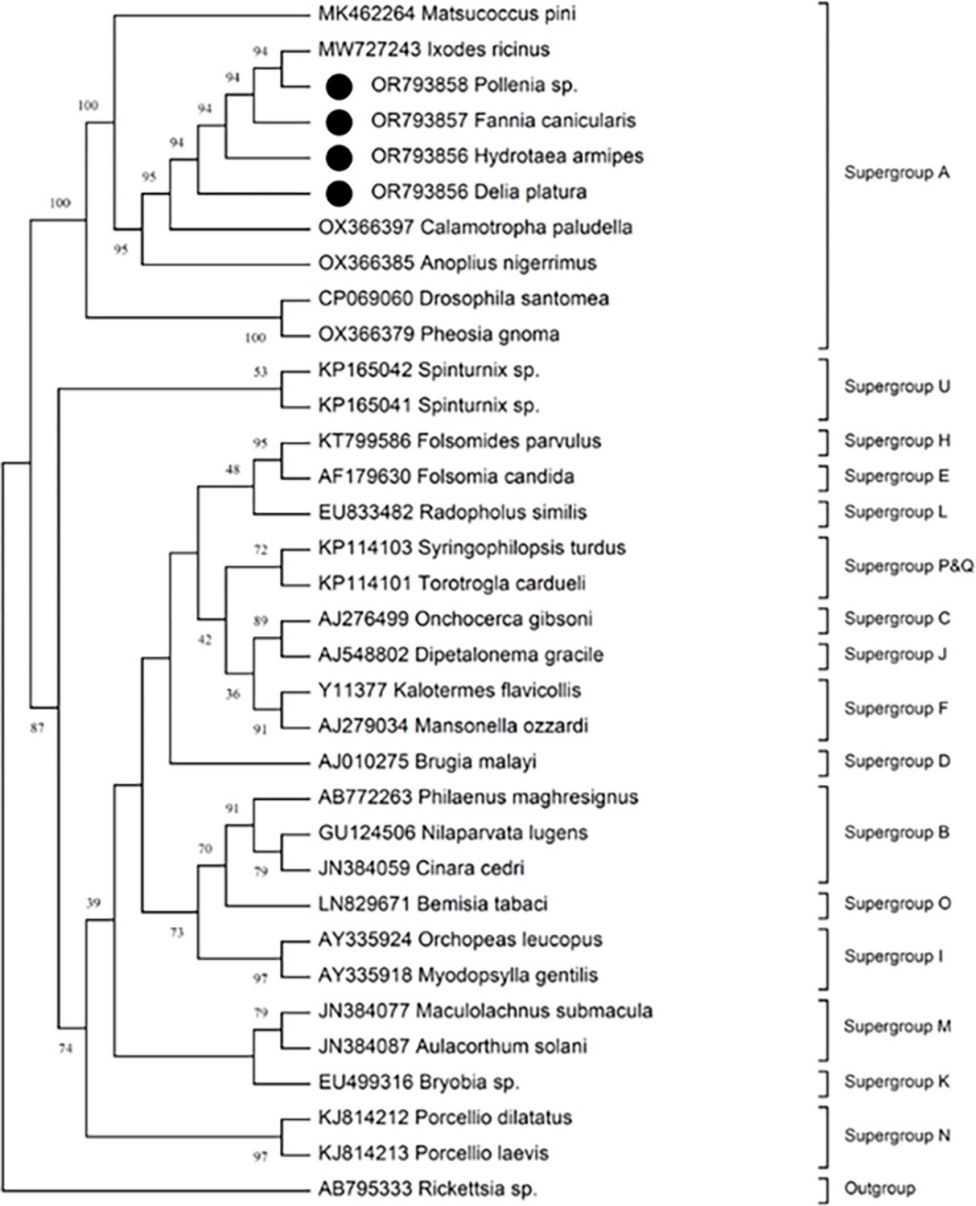
*16s rRNA* gene phylogeny based on 300–400 bp of 31 *Wolbachia* strains from 17 supergroups, showing the position of the *Wolbachia* strains isolated from *Hydrotaea armipes*, *Fannia canicularis*, *Pollenia* sp. *and Delia platura* identified in this study (black circle). Evolutionary analysis was conducted on 1000 bootstrap replications [85], using the Neighbor-Joining method [86]. Evolutionary analyses were conducted in MEGA7 [66]. GenBank ID, host species, and supergroup are indicated against the strains. The bootstrap values are shown in percentages on the internal nodes. *Rickettsia* sp. was used as an outgroup.

To confirm the Blast search results, phylogenetic analysis was performed using the sequences of *Wolbachia 16s rRNA*, *wsp*, and *gatB* genes obtained from this study, in combination with the *16s rRNA*, *wsp*, and *gatB* representatives of various *Wolbachia* supergroups retrieved from the GenBank database. All the phylogenetic analysis inferred from the three genes showed that the *Wolbachia* strains infecting the Iranian Brachycera flies were clustered with the A supergroup. The phylogenetic tree inferred from the *16s rRNA* gene is shown in Fig 4. The *16s rRNA* gene is the most important marker in identifying and characterizing new species or strains of bacteria and has been widely used in *Wolbachia* species determination and identification [76, 84]. This analysis showed that the *Wolbachia* strains infecting the Iranian Brachycera flies are associated with the strains of supergroup A and are quite distinct from the other 16 supergroups compared in this analysis.

*Wolbachia* strain MLST analysis showed that the *Wolbachia* strain detected in *H*. *armipes* matched to *Wolbachia* strains of supergroup A hosted by 15 different insect species across 13 different genera, mostly from Hymenoptera (ants) and Lepidoptera orders. Same analysis against the *gatB* sequences from *H*. *armipes*, *Pollenia sp*., and *F*. *canicularis* provided evidence for presence of similar *Wolbachia* strains of supergroup A in three ant species of *Pheidole obtusospinosa*, *Azteca* sp., and *Wasmannia* sp. all belonged to Formicidae (Hymenoptera). No *Wolbachia* MLST sequences with definite supergroup were available for the remaining (*coxA*, *gltA*, *groEL*, and *dnaA*) gene sequences.

## Discussion

This is the first report on *Wolbachia* infection in Iranian Brachycera flies. Here, we used a standard PCR technique and showed that the infection rate in Iranian fly species is about 27.3%, which is consistent with previous reports estimating that at least 20% of all insect species are infected with *Wolbachia* [21, 87]. Those estimates resulted from numerous *Wolbachia* screens, using standard PCR techniques, in which several species were tested for infection. Another study, reporting much higher infection rates (76%) [37], used a ‘long PCR’ method that is more sensitive to low concentrations of *Wolbachia* molecules. A later report by Hilgenboecker et al (2008) estimated that strains of the genus *Wolbachia* are naturally present in 66% of all insect species worldwide [87], which is more than twice what we found in the Iranian Brachyceran fly species. However, in the systematic review by Inácio da Silva et al on mosquitoes of the Order Diptera, *Wolbachia* was detected in only 30% of the mosquito species investigated [88], which agrees with our finding.

Discrepancies between reported infection rates in insects are, mainly or partly, a function of the number of specimens screened for *Wolbachia*. The problem arises in studies using a distinct species, or an only a few individuals per species. If a single specimen is infected, the species is rightly categorized as infected. However, if only one, or a few, uninfected individuals are tested, this can result in this species being defined as uninfected. The problem increases when infection rates are low, as the likelihood of detecting infection in this species would clearly have been low if only an individual specimen had been examined. It has been suggested that studies in which more than 100 individuals per species are tested incline to be biased towards infected species [87]. The sample size of some of the species screened in this study was high enough to detect *Wolbachia*; for example in *M*. *domestica* (475 specimens) and *C*. *vicinia* (180 specimens) more than 100 specimens were analysed, while *L*. *sericata* (n = 67), *Sarcophaga* sp. (n = 49), *Ch*. *albiceps* (n = 43) *Muscina stabulans* (n = 17), *Ch*. *marginalis* (n = 14), and *Ch*. *megacephala* (n = 13) numbered between 10–100 specimens. Although insect species differs in *Wolbachia* infection status and frequency across space and time, in this study, in most cases the specimens were collected from different geographical regions (Table 1). Therefore, the lack of *Wolbachia* detection in some species probably reflects the small sample sizes for these species, rather than the absence of *Wolbachia*.

In this study, we did not detect *Wolbachia* infection in certain species of the Muscidae family such as *M*. *domestica*, *Mu*. *stabulans*, and *St*. *calcitrans*. However, some species, such as *M*. *domestica*, had previously been found to be *Wolbachia*-positive by studies performed in Iran, Thailand, USA, Singapore, Denmark, and Brazil [3, 89–91], and *Wolbachia*-negative by others in different geographical regions [92], suggesting a variable infection rate and/or limitations of the *Wolbachia* detection methods employed. In this study, we were not able to study connection between the time of collection of samples because all the fly specimens were collected in warm seasons (spring or summer) when flies are active, however, no obvious connection were observed between *Wolbachia* infection and geographic origin.

All the *Wolbachia* strains detected in the Iranian flies belong to supergroup A, which, together with supergroup B, is the most common supergroup found in arthropods [80]. Interestingly, the *16s rRNA* gene sequences of *Wolbachia* strains were found in four diverse species *D*. *platura*, *H*. *armipes*, *F*. *canicularis*, and *Pollenia sp*. (except for one DNA singleton mutation) and the *gatB* gene sequences in three species *H*. *armipes*, *Pollenia* sp., and *F*. *canicularis* were identical. The presence of a single supergroup in different fly species suggests a pattern of horizontal transmission. Supergroup A has been detected in several different dipteran species, including members of the Muscidae family: *Hydrotea spinigena* [3], *Hydrotea aenescens* [93], *Musca autumnalis* [94], *Haematobia irritans irritans* [38, 92, 95]; members of the Calliphoridae family: *Protocalliphora sialia* and *Pollenia rudis* [96, 97]; members of the Fannidae: *Fannia canicularis*, *F*. *scalaris* and *F*. *pusio* [93]; member of the Glossinidae: *Glossina spp*. [98, 99], and a member of the Sarcophagidae family: *Sarcophaga dux* [3]. In addition to supergroup A, other *Wolbachia* supergroups have been reported in dipteran insects, for example supergroups A and B in sand flies [22]; A, B, C, D, and F in mosquitoes [48, 88]; A and B in *Sarcophaga* spp. [3]; A, B, and F in Hippoboscidae [100]; and A and B in *Culicoides* spp. (Ceratopogonidae) [101].

Various molecular techniques, such as PCR with specific and/or degenerate primers, PCR with various markers such as multilocus sequencing Typing (MLST), quantitative PCR (qPCR), metabarcoding, Loop Mediated Isothermal Amplification (LAMP), and Next Generation Sequencing (NGS), have been used for *Wolbachia* detection: each method shows variable specificity and sensitivity and each has certain advantages and limitations that can influence the detection of different *Wolbachia*-derived molecules and/or a true *Wolbachia* infection [22, 44, 68, 102, 103]. In this study, conventional PCR was used, with nine primer sets against nine different target genes of *Wolbachia* genome. Our results show that using various loci increases the chance of *Wolbachia* detection in the specimens. Also, using several target genes avoids the problem of integration of *Wolbachia* genes into the host’s genome, and provides a true measure of *Wolbachia* infection [99]. Consequently, we recommend that researchers should precisely select more than one method, because the evaluation of *Wolbachia* using a single method limits the inference of exact *Wolbachia* infection rates. However, compared with conventional PCR, qPCR and NGS techniques are more expensive.

Although *Wolbachia* infection has been reported in some species of Calliphoridae and Sarcophagidae, the results of this study, and available data in the literature [90, 104], show that the species of these two families are not, or are only rarely, infected with *Wolbachia*. Here, we showed that only one species (*Pollenia* sp.), out of six screened species of Calliphoridae, was infected with *Wolbachia*, and none of the six screened species of Sarcophagidae were infected. In contrast, based on the results of this study and other searches, species of the Fannidae family are usually found to be infected with *Wolbachia* [93].

Results revealed *Wolbachia* infection in six species of *Fannia canicularis*, *Fannia* sp., *Hydrotaea armipes*, *Pollenia* sp., *Desmometopa varipalpis*, and *Delia platura*. Some of these species are potential vectors for several important animal and human pathogens like viruses and bacteria, intermediate hosts of an eye worm, or have been associated with myiasis in humans [105, 106 and references herein]. However, further studies need to find any connection between the pathogens particularly the viruses and the *Wolbachia* strains found in these species.

Results of this study showed that most medically important brachyceran flies such as *M*. *domestica* were uninfected with *Wolbachia*. However, it is possible to establish stable *Wolbachia* infections in wild uninfected flies through trans-infection and thereby selectively infecting uninfected flies with specific *Wolbachia* strains and releasing infected males to the entire population. It could make flies sterile, and infertile, with reduced longevity leading to suppress population [107]. Moreover, it seems the presence of *Wolbachia* in a target insect species does not hinder control efforts because the natural infection can be either removed by antibiotic treatment (46) or kept making co-infected host [108]. Currently, three strains of *Wolbachia* (*w*AlbB, *w*Mel and *w*MelPop) have been successfully injected into adults and pupae of Buffalo flies (*Haematobia irritans exigua*) and the closely-related horn flies (*Haematobia irritans irritans*). The bacterially-induced reduced fitness, such as decreased and delayed adult emergence, reduced longevity, and reduced fecundity in *Wolbachia*-infected flies, suggest the potential of the *w*Mel or *w*MelPop strains for use in *Wolbachia*-based biocontrol programs for suppressing Buffalo fly populations [109]. It is worth mentioning that high fitness costs for released males are detrimental to insect control with *Wolbachia*. Alam et al. [110] showed that *Wolbachia* caused strong CI in *Wolbachia*-free females of tsetse flies when mated with *Wolbachia*-infected males. *Wolbachia w*Mel and *w*AlbA strains of supergroup A can induce strong CI in mosquitoes [111]. The *Wolbachia* strains that were found in this study belong to supergroup A and could be used as biocontrol agents to reduce fly-borne diseases if the strains could induce strong CI in target species. *Wolbachia* strain can be transinfected to new host by embryo microinjection [112, 113]. The suppression of harmful fly populations through CI induced by the bacterium has been used to suppress the populations of a few medically important mosquitoes such *Aedes aegypti* in USA [114] and *Aedes albopictus* in China [115].

## Conclusion

The results of this study showed that, on average, the rate of *Wolbachia* infection in the Iranian Brachycera flies is 27.3%. Also, gene sequence-based similarity analysis and phylogenetic study suggest that these flies harbor only *Wolbachia* supergroup A. The study’s limitations include detection method, sample size for some species, and lack of complete spatial and temporal sampling. More comprehensive sampling over time and space helps increase diversity of brachyceran fly species, and better view of their infections to *Wolbachia*. Such data will be fundamental to developing and employing diverse strategies that use *Wolbachia* to suppress fly populations and/or to decrease the public health burden of different fly-borne pathogens.

## Supporting information

S1 Fig The *16s rRNA* gene sequences of *Wolbachia* strains used for phylogenetic analysis in this study. (TXT)

S2 Fig The alignment *16s rRNA* gene sequences of *Wolbachia* strains used for phylogenetic analysis in this study. (TXT)

## References

[1] Frisch V, Fuehrer HP, Cavalleri JV. Relevant Brachycera (Excluding Oestroidea) for Horses in Veterinary Medicine: A Systematic Review. Pathogens. 2023;12(4):568. doi: 10.3390/pathogens12040568 37111454 PMC10142728

[2] Geden CJ, Nayduch D, Scott JG, Burgess ER, Gerry AC, Kaufman PE, et al. House Fly (Diptera: Muscidae): Biology, Pest Status, Current Management Prospects, and Research Needs. J. Integr. Pest Manag. 2021;12:39. doi: 10.1093/jipm/pmaa021

[3] Mingchay P, Sai-Ngam A, Phumee A, Bhakdeenuan P, Lorlertthum K, Thavara U, et al. *Wolbachia* supergroups A and B in natural populations of medically important filth flies (Diptera: Muscidae, Calliphoridae, and Sarcophagidae) in Thailand. Sout. As. J Trop. Med. Pub. Health. 2014;45(2):309. 24968670

[4] Akbarzadeh K, Saghafipour A, Jesri N, Karami-Jooshin M, Arzamani K, Hazratian T, et al. Spatial distribution of necrophagous flies of infraorder muscomorpha in Iran using geographical information system. J. Med. Entomol. 2018; 55(5): 1071–1085. doi: 10.1093/jme/tjy098 29982597

[5] Balaraman V, Drolet BS, Mitzel DN, Wilson WC, Owens J, Gaudreault NN, et al. Mechanical transmission of SARS-CoV-2 by house flies. Parasit. Vector. 2021 Dec; 14:1–9. doi: 10.1186/s13071-021-04703-8 33879234 PMC8056201

[6] Akbarzadeh K, Wallman JF, Sulakova H, Szpila K. Species identification of Middle Eastern blowflies (Diptera: Calliphoridae) of forensic importance. Parasitol. Res. 2015;114(4):1463–1472. doi: 10.1007/s00436-015-4329-y 25682434 PMC4365180

[7] Sarwar M. Typical Flies: Natural History, Lifestyle and Diversity of Diptera [Internet]. Life Cycle and Development of Diptera. IntechOpen; 2020. Available from: 10.5772/intechopen.91391

[8] Azarmi S, Akbarzadeh K, Ekrami A, Sheikh Z, Dehghan O. Scalp myiasis associated with soft tissue sarcoma lesion: a case report and review of relevant literature. BMC Infect Dis. 2024;24(1):51. doi: 10.1186/s12879-023-08957-8 38183025 PMC10770951

[9] Salimi M, Edalat H, Jourabchi A, Oshaghi M. First report of human nasal myiasis caused by *Eristalis tenax* in Iran (Diptera: Syrphidae). Iranian Arthropod-Borne Dis. 2010; 4(1):77–80. PMC3385543 22808393

[10] Rodríguez-Ruiz MT, Acosta AM, Cifuentes-Cardozo E, Chirveches MA, Rosselli D. Otomyiasis: Systematic Review. Int Arch Otorhinolaryngol. 2019;23(1):104–109. doi: 10.1055/s-0037-1617427 30647793 PMC6331295

[11] Moreira LA, Iturbe-Ormaetxe I, Jeffery JA, Lu G, Pyke AT, Hedges LM, et al. A *Wolbachia* symbiont in *Aedes aegypti* limits infection with dengue, Chikungunya, and *Plasmodium*. Cell. 2009; 139(7):1268–78 10.1016/j.cell.2009.11.042 20064373

[12] Guo Y, Shao J, Wu Y, Li Y. Using *Wolbachia* to control rice planthopper populations: progress and challenges. Front Microbiol. 2023; 14:1244239. doi: 10.3389/fmicb.2023.1244239 37779725 PMC10537216

[13] Gong JT, Li TP, Wang MK, Hong XY. *Wolbachia*-based strategies for control of agricultural pests. Curr. Opin. Insect Sci. 2023; 57:101039. doi: 10.1016/j.cois.2023.101039 37105498

[14] Mateos M, Martinez Montoya H, Lanzavecchia SB, Conte C, Guillén K, Morán-Aceves BM, et al. *Wolbachia pipientis* Associated with *Tephritid* Fruit Fly Pests: From Basic Research to Applications. Front Microbiol. 2020;11:1080. doi: 10.3389/fmicb.2020.01080 32582067 PMC7283806

[15] Atyame CM, Labbe P, Dumas E, Milesi P, Charlat S, Fort P, et al. *Wolbachia* divergence and the evolution of cytoplasmic incompatibility in *Culex pipiens*. PloS One. 2014;9(1):e87336. 24498078 10.1371/journal.pone.0087336 PMC3909092

[16] Hunter MS, Perlman SJ, Kelly SE. A bacterial symbiont in the Bacteroidetes induces cytoplasmic incompatibility in the parasitoid wasp *Encarsia pergandiella*. Proceedings of the Royal Society of London Series B: Biological Sciences. 2003;270(1529):2185–90. 10.1098/rspb.2003.2475 PMC1691482 14561283

[17] Kittayapong P, Ninphanomchai S, Limohpasmanee W, Chansang C, Chansang U, Mongkalangoon P. Combined sterile insect technique and incompatible insect technique: The first proof-of-concept to suppress *Aedes aegypti* vector populations in semi-rural settings in Thailand. PLoS Neg. Trop. Dis. 2019;13(10):e0007771. 10.1371/journal.pntd.0007771 PMC6837763 31658265

[18] Werren JH, Baldo L, Clark ME. *Wolbachia*: master manipulators of invertebrate biology. Nat. Rev. Mic. 2008;6(10):741–51. 10.1038/nrmicro1969 18794912

[19] Riegler M, Stauffer C. *Wolbachia* infections and superinfections in cytoplasmically incompatible populations of the European cherry fruit fly *Rhagoletis cerasi* (Diptera, Tephritidae). Mol. Eco. 2002; 11(11):2425–34. 10.1098/rspb.1998.0324 12406252

[20] Zhou W, Rousset F, O’Neill S. Phylogeny and PCR–based classification of *Wolbachia* strains using *wsp* gene sequences. Proc. Biol. Sci. 1998; 265(1395):509–13. 10.1098/rspb.1998.0324 9569669 PMC1688917

[21] Werren JH, Windsor DM. *Wolbachia* infection frequencies in insects: evidence of a global equilibrium? Proc. Bio. Sci. 2000;267(1450):1277–85. 10.1098/rspb.2000.1139 10972121 PMC1690679

[22] Karimian F, Vatandoost H, Rassi Y, Maleki-Ravasan N, Choubdar N, Koosha M, et al. *wsp*-based analysis of *Wolbachia* strains associated with *Phlebotomus papatasi* and *P*. *sergenti* (Diptera: Psychodidae) main cutaneous leishmaniasis vectors, introduction of a new subgroup *w*Serg. Patho. Glob. Health. 2018; 112(3):152–60. 10.1080/20477724.2018.1471438 PMC6056827 29745300

[23] Stewart FJ, Newton IL, Cavanaugh CM. Chemosynthetic endosymbioses: adaptations to oxic–anoxic interfaces. Trends Microbiol. 2005; 13(9):439–48. doi: 10.1016/j.tim.2005.07.007 16054816

[24] Nair S. Bacterial Associations: Antagonism to Symbiosis. Nat. Inst. Oceano. Goa; 2004.

[25] Vautrin E, Vavre F. Interactions between vertically transmitted symbionts: cooperation or conflict? Trends Microbiol. 2009;17(3):95–9. doi: 10.1016/j.tim.2008.12.002 19230673

[26] Taillardat-Bisch A-V, Raoult D, Drancourt M. RNA polymerase β-subunit-based phylogeny of *Ehrlichia* spp., *Anaplasma* spp., *Neorickettsia* spp. and *Wolbachia pipientis*. Int. J. Syst. Evol. Microbiol. 2003; 53(2):455–8. 10.1099/ijs.0.02411-0 12710612

[27] Gomes TM, Wallau GL, Loreto EL. Multiple long-range host shifts of major *Wolbachia* supergroups infecting arthropods. Sci. Rep. 2022; 12(1):8131. 10.1038/s41598-022-12299-x 35581290 PMC9114371

[28] Sharma AK, Som A. Assigning new supergroups V and W to the *Wolbachia* diversity. Bioinformation. 2023; 19(3):336–340. 10.6026/97320630019336. 37808371 PMC10557451

[29] Hertig M, Wolbach SB. Studies on rickettsia-like micro-organisms in insects. J. Med. Res. 1924;44(3):329. 19972605 PMC2041761

[30] Weeks AR, Breeuwer JA. *Wolbachia*–induced parthenogenesis in a genus of phytophagous mites. Proc. Royal Soc. B. Bio. Sci. 2001; 268(1482):2245–51. doi: 10.1098/rspb.2001.1797 11674872 PMC1088872

[31] Rousset F, Bouchon D, Pintureau B, Juchault P, Solignac M. *Wolbachia* endosymbionts responsible for various alterations of sexuality in arthropods. Proc. Bio. Sci. 1992; 250(1328):91–8. doi: 10.1098/rspb.1992.0135 1361987

[32] Sharma G, Malthankar PA, Mathur V. Insect–plant interactions: a multilayered relationship. Annal. Entomo. Soci. Amer. 2021 Jan 1;114(1):1–6.

[33] Charlat S, Duplouy A, Hornett EA, Dyson EA, Davies N, Roderick GK, et al. The joint evolutionary histories of *Wolbachia* and mitochondria in *Hypolimnas bolina*. BMC Evo. Bio. 2009; 9(1):1–9. 10.1186/1471-2148-9-64. PMC2669805 19317891

[34] Kaur R, Shropshire JD, Cross KL, Leigh B, Mansueto AJ, Stewart V, et al. Living in the endosymbiotic world of *Wolbachia*: a centennial review. Cell Host. Microbe. 2021; 29(6):879–93. 10.1016/j.chom.2021.03.006. 33945798 PMC8192442

[35] Marcon HS, Coscrato VE, Selivon D, Perondini AL, Marino CL. Variations in the sensitivity of different primers for detecting *Wolbachia* in *Anastrepha* (Diptera: Tephritidae). Braz J Microbiol. 2011; 42(2):778–85. doi: 10.1590/S1517-838220110002000046 24031693 PMC3769819

[36] Li Y, Sun Y, Zou J, Zhong D, Liu R, Zhu C, Li W, Zhou Y, Cui L, Zhou G, Lu G, Li T. Characterizing the *Wolbachia* infection in field-collected Culicidae mosquitoes from Hainan Province, China. Parasit Vectors. 2023; 16(1):128. doi: 10.1186/s13071-023-05719-y 37060070 PMC10103416

[37] Jeyaprakash A., Hoy M.A. Long PCR improves *Wolbachia* DNA amplification: *wsp* sequences found in 76% of sixty-three arthropod species. Insect Mol. Biol. 2000; 9:393–405. 10971717 10.1046/j.1365-2583.2000.00203.x

[38] Xiao-Yue H., Gotoh T., Noda H. Sensitivity comparison of PCR primers for detecting *Wolbachia* in spider mites. Appl. Entomol. Zool. 2002; 37:379–383.

[39] Jamnongluk W., Kittayapong P., Baimai V., O’Neill. S.L. *Wolbachia* infections of tephritid fruit flies: molecular evidence for five distinct strains in a single host species. Curr. Microbiol. 2002; 45:255–260. 12192522 10.1007/s00284-002-3746-1

[40] Baldo L., Bordenstein S., Wernegreen J. J., and Werren J. H. Widespread recombination throughout *Wolbachia* genomes. Mol. Biol. Evol. 2006; 23:437–449. 16267140 10.1093/molbev/msj049

[41] Baldo L, Dunning Hotopp JC, Jolley KA, Bordenstein SR, Biber SA, Choudhury RR, Hayashi C, Maiden MC, Tettelin H, Werren JH. Multilocus sequence typing system for the endosymbiont *Wolbachia pipientis*. Appl. Environ. Microbiol. 2006; 72(11):7098–110. doi: 10.1128/AEM.00731-06 16936055 PMC1636189

[42] Kajtoch Ł, Kotásková N. Current state of knowledge on *Wolbachia* infection among Coleoptera: a systematic review. PeerJ. 2018; 6:e4471. 10.7717/peerj.4471. 29568706 PMC5846457

[43] Karimian F, Koosha M, Choubdar N, Oshaghi MA. Comparative analysis of the gut microbiota of sand fly vectors of zoonotic visceral leishmaniasis (ZVL) in Iran; host-environment interplay shapes diversity. PLoS Negl. Trop. Dis. 2022; 16(7):e0010609. doi: 10.1371/journal.pntd.0010609 35853080 PMC9337680

[44] Bordbar A, Soleimani S, Fardid F, Zolfaghari MR, Parvizi P. Three strains of *Wolbachia pipientis* and high rates of infection in Iranian sandfly species. Bull. Entomo. Res. 2014; 104(2):195–202. https://doi.10.1017/S0007485313000631. 10.1017/S0007485313000631 24484966

[45] Parvizi P, Bordbar A, Najafzadeh N. Detection of *Wolbachia pipientis*, including a new strain containing the *wsp* gene, in two sister species of Paraphlebotomus sandflies, potential vectors of zoonotic cutaneous leishmaniasis. Mem Inst Oswaldo Cruz. 2013; 108:414–20. doi: 10.1590/0074-0276108042013004 . 23828002 PMC3970627

[46] Parvizi P, Fardid F, Soleimani S. Detection of a new strain of *Wolbachia pipientis* in *Phlebotomus perfiliewi transcaucasicus*, a potential vector of visceral leishmaniasis in North West of Iran, by targeting the major surface protein gene. J Arthropod-Borne Dis. 2013; 7(1):46. 23785694 PMC3684496

[47] Choubdar N, Karimian F, Koosha M, Nejati J, Shabani Kordshouli R, Azarm A, Oshaghi MA. *Wolbachia* infection in native populations of *Blattella germanica* and *Periplaneta americana*. PLoS One. 2023; 18(4):e0284704. doi: 10.1371/journal.pone.0284704 37079598 PMC10118093

[48] Karami M, Moosa-Kazemi SH, Oshaghi MA, Vatandoost H, Sedaghat MM, Rajabnia R, et al. *Wolbachia* endobacteria in natural populations of *Culex pipiens* of Iran and its phylogenetic congruence. J Arthropod-borne Dis. 2016; 10(3):347. 27308293 PMC4906741

[49] Shemshadian A, Vatandoost H, Oshaghi MA, Abai MR, Djadid ND. Relationship between *Wolbachia* infection in *Culex quinquefasciatus* and its resistance to insecticide. Heliyon. 2021; 7(4): e06749. doi: 10.1016/j.heliyon.2021.e06749 33912718 PMC8065290

[50] Maleki‐Ravasan N, Akhavan N, Raz A, Jafari M, Zakeri S, Dinparast Djadid N. Co‐occurrence of pederin‐producing and *Wolbachia* endobacteria in *Paederus fuscipes* Curtis, 1840 (Coleoptera: Staphilinidae) and its evolutionary consequences. Microbiology Open. 2019; 8(7):e00777. https://doi.10.1002/mbo3.777. 30560551 10.1002/mbo3.777 PMC6612549

[51] Chamankar B, Maleki-Ravasan N, Karami M, Forouzan E, Karimian F, Naeimi S, et al. The structure and diversity of microbial communities in *Paederus fuscipes* (Coleoptera: Staphylinidae): from ecological paradigm to pathobiome. Microb. 2023; 11(1):1–20. 10.1186/s40168-022-01456-z PMC9862579 36670494

[52] Akbarzadeh K, Rafinejad J, Nozari J, Rassi Y, Sedaghat MM, Hosseini M. A modified trap for adult sampling of medically important flies (Insecta: Diptera). J Arthropod Borne Dis. 2012; 6(2):119–28. 23378969 PMC3547306

[53] Crosskey RW, Lane RP. House-flies, blow-flies and their allies (calyptrate Diptera). Medical Insects and Arachnids (ed. by Lane R. P and Crosskey R. W), (1993) pp. 403–428. Chapman & Hall, London.

[54] James MT. Family Stratiomyidae, vol. 26. In: A catalogue of the Diptera of the Americas South of the United States. 1973, São Paulo: Museu de Zoologia, Universidade de São Paulo.

[55] Zumpt F. Myiasis in Man and Animals of the World. A textbook for Physicians and veterinarians and zoologist. 1965. London. Butterworthe.Xv, + 267 pp.

[56] Parchami-Araghi M, Peris SV, Gonzáles-Mora D. New records of Iranian Calliphoridae and Sarcophagidae, with a guide to the males of Palaearctic Protocalliphora (Diptera, Calyptratae). Bol. R. Soc. Esp. Hist. Nat. (Sec. Biol.) 2001;96:175–181.

[57] Collins FH, Mendez MA, Rasmussen MO, Mehaffey PC, Besansky NJ, Finnerty V. A ribosomal RNA gene probe differentiates member species of the *Anopheles gambiae* complex. Am J Trop Med Hyg. 1987;37(1):37–41. doi: 10.4269/ajtmh.1987.37.37 2886070

[58] Koosha M, Oshaghi MA, Sedaghat MM, Vatandoost H, Azari-Hamidian S, Abai MR, Hanafi-Bojd AA, Mohtarami F. Sequence analysis of mtDNA COI barcode region revealed three haplotypes within *Culex pipiens* assemblage. Exp Parasitol. 2017; 181:102–110. doi: 10.1016/j.exppara.2017.08.003 28818649

[59] Braig HR, Zhou W, Dobson SL, O’Neill SL. Cloning and characterization of a gene encoding the major surface protein of the bacterial endosymbiont *Wolbachia pipientis*. Bacteriol. 1998;180(9):2373–8. https://doi.10.1128/JB.180.9.2373–2378.1998. 10.1128/jb.180.9.2373-2378.1998 PMC107178 9573188

[60] Jiggins FM. The rate of recombination in *Wolbachia* bacteria. Mol. Biol. Evo.2002; 19(9):1640–3. https://doi.10.1093/oxfordjournals.molbev.a004228. 10.1093/oxfordjournals.molbev.a004228 12200493

[61] http://www.technelysium.com.au/chromas.html

[62] https://www.ncbi.nlm.nih.gov/

[63] https://www.ebi.ac.uk/Tools/msa/clustalo

[64] Sievers F, Wilm A, Dineen D, Gibson TJ, Karplus K, Li W, Lopez R, McWilliam H, Remmert M, Söding J, Thompson JD, Higgins DG. Fast, scalable generation of high-quality protein multiple sequence alignments using Clustal Omega. Mol Syst Biol. 2011; 7:539. doi: 10.1038/msb.2011.75 21988835 PMC3261699

[65] Nishimaki T, Sato K. An Extension of the Kimura Two-Parameter Model to the Natural Evolutionary Process. J. Mol. Evol. 2019; 87(1):60–67. doi: 10.1007/s00239-018-9885-1 30631891 PMC6514111

[66] Kumar S, Stecher G, Tamura K. MEGA7: Molecular evolutionary genetics analysis version 7.0 for bigger datasets. Mol. Bio. Evo. 2016; 33(7):1870–4. doi: 10.1093/molbev/msw054 27004904 PMC8210823

[67] Werren JH, Zhang W, Guo LR. Evolution and phylogeny of *Wolbachia*: reproductive parasites of arthropods. Proc. Bio. Sci. 1995; 261(1360):55–63. 10.1098/rspb.1995.0117. 7644549

[68] Casiraghi M, Bordenstein S, Baldo L, Lo N, Beninati T, Wernegreen J, et al. Phylogeny of *Wolbachia pipientis* based on *gltA*, *groEL* and *ftsZ* gene sequences: clustering of arthropod and nematode symbionts in the F supergroup, and evidence for further diversity in the *Wolbachia* tree. Microbiology (Reading). 2005;151(Pt 12):4015–4022. doi: 10.1099/mic.0.28313-0.Mic 16339946

[69] Jeffries CL, Tantely LM, Raharimalala FN, Hurn E, Boyer S, Walker T. Diverse novel resident *Wolbachia* strains in Culicine mosquitoes from Madagascar. Sci. Rep. 2018; 8(1):1–15. 10.1038/s41598-018-35658-z 30498246 PMC6265278

[70] Lo N, Casiraghi M, Salati E, Bazzocchi C, Bandi C. How many *Wolbachia* supergroups exist? Mol. Biol Evo. 2002; 19(3):341–6. 10.1093/oxfordjournals.molbev.a004087 11861893

[71] Jiggins FM. The rate of recombination in *Wolbachia* bacteria. Mol. Biol. Evo. 2002; 19(9):1640–3. 10.1093/oxfordjournals.molbev.a004228 12200493

[72] Augustinos AA, Santos-Garcia D, Dionyssopoulou E, Moreira M, Papapanagiotou A, Scarvelakis M, Doudoumis V, Ramos S, Aguiar AF, Borges PA, Khadem M, Latorre A, Tsiamis G, Bourtzis K. Detection and characterization of *Wolbachia* infections in natural populations of aphids: is the hidden diversity fully unraveled? PLoS One. 2011; 6(12):e28695. doi: 10.1371/journal.pone.0028695 22174869 PMC3236762

[73] Wang WX, Zhu TH, Lai FX, Fu Q. Diversity and infection frequency of symbiotic bacteria in different populations of the rice brown planthopper in China. J. Entomol. Sci. 2015; 50(1):47–66. 10.18474/0749-8004-50.1.47

[74] Koga R, Bennett GM, Cryan JR, Moran NA. Evolutionary replacement of obligate symbionts in an ancient and diverse insect lineage. Environ Microbiol. 2013; 15(7):2073–81. doi: 10.1111/1462-2920.12121 23574391

[75] Bandi C, Anderson TJ, Genchi C, Blaxter ML. Phylogeny of *Wolbachia* in filarial nematodes. Proc Biol Sci. 1998; 265(1413):2407–13. doi: 10.1098/rspb.1998.0591 9921679 PMC1689538

[76] Vandekerckhove TT, Watteyne S, Willems A, Swings JG, Mertens J, Gillis M. Phylogenetic analysis of the 16s rRNA of the cytoplasmic bacterium *Wolbachia* from the novel host *Folsomia candida* (Hexapoda, Collembola) and its implications for Wolbachial taxonomy. FEMS Microbiol Lett. 1999;180(2):279–86. doi: 10.1111/j.1574-6968.1999.tb08807.x 10556723

[77] Ma Y, Chen WJ, Li ZH, Zhang F, Gao Y, Luan YX. Revisiting the phylogeny of *Wolbachia* in Collembola. Ecol Evol. 2017; 7(7):2009–2017. doi: 10.1002/ece3.2738 28405268 PMC5383468

[78] Dittmar K, Whiting MF. New *Wolbachia* endosymbionts from Nearctic and Neotropical fleas (Siphonaptera). J Parasitol. 2004; 90(5):953–7. doi: 10.1645/GE-186R 15562592

[79] Casiraghi M, Bain O, Guerrero R, Martin C, Pocacqua V, Gardner SL, Franceschi A, Bandi C. Mapping the presence of *Wolbachia pipientis* on the phylogeny of filarial nematodes: evidence for symbiont loss during evolution. Int J Parasitol. 2004; 34(2):191–203. doi: 10.1016/j.ijpara.2003.10.004 15037105

[80] Ros VI, Fleming VM, Feil EJ, Breeuwer JA. How diverse is the genus *Wolbachia*? Multiple-gene sequencing reveals a putatively new *Wolbachia* supergroup recovered from spider mites (Acari: Tetranychidae). Appl Environ Microbiol. 2009; 75(4):1036–43. doi: 10.1128/AEM.01109-08 19098217 PMC2643572

[81] Zimmermann BL, Bouchon D, Almerão MP, Araujo PB. *Wolbachia* in Neotropical terrestrial isopods. FEMS Microbiol Ecol. 2015; 91(4):fiv025. doi: 10.1093/femsec/fiv025 25764472

[82] Glowska E, Dragun-Damian A, Dabert M, Gerth M. New *Wolbachia* supergroups detected in quill mites (Acari: Syringophilidae). Infect. Genet. Evol. 2015; 30:140–6. 10.1016/j.meegid.2014.12.019 25541519

[83] Ishii Y, Matsuura Y, Kakizawa S, Nikoh N, Fukatsu T. Diversity of bacterial endosymbionts associated with *Macrosteles* leafhoppers vectoring phytopathogenic phytoplasmas. Appl Environ Microbiol. 2013;79(16):5013–22. doi: 10.1128/AEM.01527-13 23770905 PMC3754707

[84] Hassler HB, Probert B, Moore C, Lawson E, Jackson RW, Russell BT, et al. Phylogenies of the *16s rRNA* gene and its hypervariable regions lack concordance with core genome phylogenies. Microbiome. 2022; 10(1):104. 10.1186/s40168-022-01295-y 35799218 PMC9264627

[85] Felsenstein J. Confidence limits on phylogenies: an approach using the bootstrap. Evolution. 1985; 39(4):783–91. doi: 10.1111/j.1558-5646.1985.tb00420.x 28561359

[86] Saitou N, Nei M. The neighbor-joining method: a new method for reconstructing phylogenetic trees. Mol. Biol. Evol. 1987; 4(4):406–25. doi: 10.1093/oxfordjournals.molbev.a040454 3447015

[87] Hilgenboecker K, Hammerstein P, Schlattmann P, Telschow A, Werren JH. How many species are infected with *Wolbachia*?–a statistical analysis of current data. FEMS Microbiol. Lett. 2008; 281(2):215–20. 10.1111/j.1574-6968.2008.01110.x 18312577 PMC2327208

[88] Inácio da Silva LM, Dezordi FZ, Paiva MHS, Wallau GL. Systematic review of *Wolbachia* symbiont detection in mosquitoes: An entangled topic about methodological power and true symbiosis. Pathogens. 2021;10(1):39. 10.3390/pathogens10010039. 33419044 PMC7825316

[89] Bahrndorff S, De Jonge N, Skovgård H, Nielsen JL. Bacterial communities associated with houseflies (Musca domestica L.) sampled within and between farms. PLoS One. 2017; 12(1):e0169753. doi: 10.1371/journal.pone.0169753 28081167 PMC5232358

[90] Pourali P, Roayaei AM, Jeloudar A, Razi JMH. PCR screening of the *Wolbachia* in some arthropods and nematodes in Khuzestan province. Iranian J. Vet. Res. 10(3): 216–222. doi: 10.22099/ijvr.2009.1695

[91] Junqueira AC, Azeredo-Espin AM, Paulo DF, Marinho MA, Tomsho LP, Drautz-Moses DI, et al. Large-scale mitogenomics enables insights into Schizophora (Diptera) radiation and population diversity. Scient. Rep. 2016; 6(1):21762. doi: 10.1038/srep21762 26912394 PMC4766414

[92] Floate KD, Kyei-Poku GK, Coghlin PC. Overview and relevance of *Wolbachia* bacteria in biocontrol research. Biocontrol Sci. Technol. 2006; 16(8):767–88. 10.1080/09583150600699606

[93] Grzywacz A, Wyborska D, Piwczyński M. DNA barcoding allows identification of European Fanniidae (Diptera) of forensic interest. Forensic. Sci. Int. 2017; 278:106–14. doi: 10.1016/j.forsciint.2017.06.023 28734268

[94] Kyei‐Poku G, Giladi M, Coghlin P, Mokady O, Zchori‐Fein E, Floate K. *Wolbachia* in wasps parasitic on filth flies with emphasis on *Spalangia cameroni*. Entomologia Experimentalis et Applicata. 2006; 121(2):123–35. 10.1111/j.1570-8703.2006.00469.x

[95] Torres L, Almazán C, Ayllón N, Galindo RC, Rosario-Cruz R, Quiroz-Romero H, Gortazar C, de la Fuente J. Identification of microorganisms in partially fed female horn flies, *Haematobia irritans*. Parasitol Res. 2012; 111(3):1391–5. doi: 10.1007/s00436-012-2877-y 22411632

[96] Baudry E, Bartos J, Emerson K, Whitworth T, Werren J. *Wolbachia* and genetic variability in the birdnest blowfly *Protocalliphora sialia*. Mol. Ecol. 2003; 12(7):1843–54. 10.1046/j.1365-294X.2003.01855.x 12803636

[97] Whitworth T, Dawson R, Magalon H, Baudry E. DNA barcoding cannot reliably identify species of the blowfly genus *Protocalliphora* (Diptera: Calliphoridae). Proc. Biol. Sci. 2007; 274(1619):1731–9. 10.1098/rspb.2007.0062 17472911 PMC2493573

[98] Doudoumis V, Tsiamis G, Wamwiri F, Brelsfoard C, Alam U, Aksoy E, Dalaperas S, Abd-Alla A, Ouma J, Takac P, Aksoy S, Bourtzis K. Detection and characterization of *Wolbachia* infections in laboratory and natural populations of different species of tsetse flies (genus *Glossina*). BMC Microbiol. 2012;12 Suppl 1(Suppl 1):S3. doi: 10.1186/1471-2180-12-S1-S3 22376025 PMC3287514

[99] International *Glossina* Genome Initiative. Genome sequence of the tsetse fly (*Glossina morsitans*): vector of African trypanosomiasis. Science. 2014;344(6182):380–6. doi: 10.1126/science.1249656 24763584 PMC4077534

[100] Liu Y, He B, Li F, Li K, Zhang L, Li X, et al. Molecular identification of *Bartonella melophagi* and *Wolbachia* supergroup F from sheep keds in Xinjiang, China. Korean J. Parasito. 2018; 56(4):365–370. https://doi.org/10.3347/kjp.2018.56.4.365 10.3347/kjp.2018.56.4.365 PMC6137304 30196669

[101] Pagès N, Muñoz-Muñoz F, Verdún M, Pujol N, Talavera S. First detection of *Wolbachia*-infected *Culicoides* (Diptera: Ceratopogonidae) in Europe: *Wolbachia* and *Cardinium* infection across *Culicoides* communities revealed in Spain. Parasit.Vector. 2017; 10(1),582. 10.1186/s13071-017-2486-9 PMC5701505 29169377

[102] Leggewie M, Krumkamp R, Badusche M, Heitmann A, Jansen S, Schmidt-Chanasit J, et al. *Culex torrentium* mosquitoes from Germany are negative for *Wolbachia*. Med. Vet. Entomol. 2018; 32:115–120. 10.1111/mve.12270. 28906572

[103] Balaji S, Jayachandran S, Prabagaran SR. Evidence for the natural occurrence of *Wolbachia* in *Aedes aegypti* mosquitoes. FEMS Microbiol. Lett. 2019; 366(6):fnz055. 10.1093/femsle/fnz055 30869785

[104] Șakİ CE, Șİmșek S. Investigation of *Wolbachia* spp. in some flies species (Order: Diptera) by PCR. Kafkas Üniversitesi Veteriner Fakültesi Dergisi. 2014; 20(3):417–9. https://doi.10.9775/kvfd.2013.10306.

[105] Faulde M, Sobe D, Burghardt H, Wermter R. Hospital infestation by the cluster fly, *Pollenia rudis* sensu stricto Fabricius 1794 (Diptera: Calliphoridae), and its possible role in transmission of bacterial pathogens in Germany. Int J Hyg Environ Health. 2001; 203(3):201–4. doi: 10.1078/S1438-4639(04)70029-2 11279815

[106] Murillo AC, Hubbard CB, Hinkle NC, Gerry AC. Big Problems With Little House Fly (Diptera: Fanniidae). J. Integr. Pest Manag. 2021; 12(1): 40 10.1093/jipm/pmaa023

[107] Sicard M, Bonneau M, Weill M. *Wolbachia* prevalence, diversity, and ability to induce cytoplasmic incompatibility in mosquitoes. Curr. Opin. Insect Sci. 2019; 34:12–20. doi: 10.1016/j.cois.2019.02.005 31247412

[108] Moretti R, Yen PS, Houé V, Lampazzi E, Desiderio A, Failloux AB, Calvitti M. Combining *Wolbachia*-induced sterility and virus protection to fight *Aedes albopictus*-borne viruses. PLoS Negl Trop Dis. 2018; 2(7):e0006626. doi: 10.1371/journal.pntd.0006626 30020933 PMC6066253

[109] Madhav M, Brown G, Morgan JAT, Asgari S, McGraw EA, James P. Transinfection of buffalo flies (*Haematobia irritans exigua*) with *Wolbachia* and effect on host biology. Parasit. Vector. 2020; 13(1):296. 10.1186/s13071-020-04161-8 PMC7285521 32522243

[110] Alam U, Medlock J, Brelsfoard C, Pais R, Lohs C, Balmand S, et al. *Wolbachia* symbiont infections induce strong cytoplasmic incompatibility in the tsetse fly *Glossina morsitans*. PLoS Pathog. 2011; 7(12):e1002415. doi: 10.1371/journal.ppat.1002415 22174680 PMC3234226

[111] Liang X, Liu J, Bian G, Xi Z. *Wolbachia* Inter-Strain Competition and Inhibition of Expression of Cytoplasmic Incompatibility in Mosquito. Front Microbiol. 2020 Jul 10;11:1638. doi: 10.3389/fmicb.2020.01638 32765466 PMC7381284

[112] Sasaki T, Ishikawa H. Transinfection of *Wolbachia* in the mediterranean flour moth, *Ephestia kuehniella*, by embryonic microinjection. Heredity (Edinb). 85 (Pt 2): 130–135. 11012714 10.1046/j.1365-2540.2000.00734.x

[113] Ant TH, Herd C, Louis F, Failloux AB, Sinkins SP. *Wolbachia* transinfections in *Culex quinquefasciatus* generate cytoplasmic incompatibility. Insect Mol Biol. 2020; 29(1):1–8. doi: 10.1111/imb.12604 31194893 PMC7027843

[114] Crawford JE, Clarke DW, Criswell V, Desnoyer M, Cornel D, Deegan B, et al. Efficient production of male *Wolbachia*-infected *Aedes aegypti* mosquitoes enables large-scale suppression of wild populations. Nat. Biotechnol. 2020; 38(4):482–492. doi: 10.1038/s41587-020-0471-x 32265562

[115] Zhang D, Zheng X, Xi Z, Bourtzis K, Gilles JR. Combining the sterile insect technique with the incompatible insect technique: I-impact of *Wolbachia* infection on the fitness of triple- and double-infected strains of *Aedes albopictus*. PLoS One. 2015; 10(4):e0121126. doi: 10.1371/journal.pone.0121126 25849812 PMC4388707

